# Feasibility Study of Automated Framework for Estimating Lung Tumor Locations for Target-Based Patient Positioning in Stereotactic Body Radiotherapy

**DOI:** 10.1155/2015/653974

**Published:** 2015-01-05

**Authors:** Satoshi Yoshidome, Hidetaka Arimura, Katsumasa Nakamura, Yoshiyuki Shioyama, Kazushige Atsumi, Yasuhiko Nakamura, Hideki Yoshikawa, Kei Nishikawa, Hideki Hirata

**Affiliations:** ^1^Department of Clinical Radiology, Kyushu University Beppu Hospital, 4546 Tsurumibaru, Tsurumi, Beppu 874-0838, Japan; ^2^Department of Health Sciences, Graduate School of Medical Sciences, Kyushu University, 3-1-1 Maidashi, Higashi-ku, Fukuoka 812-8582, Japan; ^3^Department of Health Sciences, Faculty of Medical Sciences, Kyushu University, 3-1-1 Maidashi, Higashi-ku, Fukuoka 812-8582, Japan; ^4^Department of Clinical Radiology, Graduate School of Medical Sciences, Kyushu University, 3-1-1 Maidashi, Higashi-ku, Fukuoka 812-8582, Japan; ^5^Department of Radiology, Kyushu University Beppu Hospital, 4546 Tsurumibaru, Tsurumi, Beppu 874-0838, Japan; ^6^Division of Radiology, Department of Medical Technology, Kyushu University Hospital, 3-1-1 Maidashi, Higashi-ku, Fukuoka 812-8582, Japan

## Abstract

*Objective.* To investigate the feasibility of an automated framework for estimating the lung tumor locations for tumor-based patient positioning with megavolt-cone-beam computed tomography (MV-CBCT) during stereotactic body radiotherapy (SBRT). *Methods.* A lung screening phantom and ten lung cancer cases with solid lung tumors, who were treated with SBRT, were employed to this study. The locations of tumors in MV-CBCT images were estimated using a tumor-template matching technique between a tumor template and the MV-CBCT. Tumor templates were produced by cropping the gross tumor volume (GTV) regions, which were enhanced by a Sobel filter or a blob structure enhancement (BSE) filter. Reference tumor locations (grand truth) were determined based on a consensus between a radiation oncologist and a medical physicist. *Results.* According to the results of the phantom study, the average Euclidean distances of the location errors in the original, Sobel-filtered, and BSE-filtered images were 2.0 ± 4.1 mm, 12.8 ± 9.4 mm, and 0.4 ± 0.5 mm, respectively. For clinical cases, these were 3.4 ± 7.1 mm, 7.2 ± 11.6 mm, and 1.6 ± 1.2 mm, respectively. *Conclusion.* The feasibility study suggests that our proposed framework based on the BSE filter may be a useful tool for tumor-based patient positioning in SBRT.

## 1. Introduction

Stereotactic body radiotherapy (SBRT) is employed for administering higher doses to ablate lung cancer while sparing surrounding normal tissues. In SBRT, a high dose of radiation, such as 12 Gy per session, is administered to a small localized region for small number of fractions [1–5]. The key to successful SBRT is the accurate patient positioning with immobilization devices and the use of image-guided positioning technologies using a two-dimensional (2D) electronic portal imaging device (EPID), 2D kilovolt- (kV-) imaging device, and/or three-dimensional (3D) kV- or megavolt- (MV-) cone-beam computed tomography (CBCT), which are employed for acquiring “verification images” [6–11].

CBCT-based image-guided radiotherapy (IGRT) systems are widely used to increase the accuracy of the SBRT. However, many institutions use the following two-step IGRT-based procedure for the patient setup, that is, (1) the initial patient positioning based on bone structures and then (2) the manual and subjective translation of patients so that tumors' regions in the verification images align with those in the planning CT images [12, 13]. This is because bone-based patient positioning leads to tumor misalignment between treatment planning CT images and pretreatment CBCT images, and it requires relatively larger safety margins because of the large interfractional baseline shifts and the deformity of the tumor [14–17]. If the patient positioning could be automatically and quantitatively performed by solely one-step procedure based on the tumor, it would result in better reproducibility of the patient setup for several fractions. Furthermore, if the tumor-based automated approach would accurately perform the patient setup, we could achieve high rates of local tumor control with low rates of toxicity [18–20] and would reduce the time required for patient positioning due to reduction of one step.

To the best of our knowledge, there have been no studies on frameworks for estimating lung tumor locations, which can be employed for automated patient positioning based on tumor regions. The goal of the present study was to investigate the feasibility of an automated framework for estimating the lung tumor locations for tumor-based patient positioning with MV-CBCT in SBRT.

## 2. Methods and Materials

### 2.1. Overall Design of Proposed Framework

**Figure 1:**
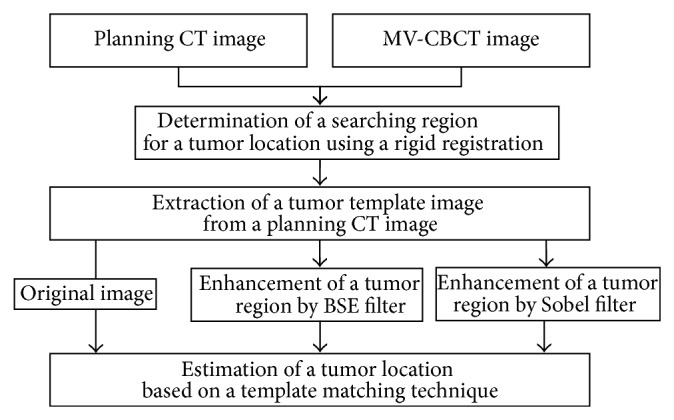
The overall design for estimating the lung tumor location in MV-CBCT images.

Figure 1 presents the overall design of the proposed framework. In the proposed framework, the tumor location in an MV-CBCT image is estimated for each fraction using a tumor-template matching technique between the tumor template and MV-CBCT image within the search region determined based on the gross tumor volume (GTV). The search region for the tumor location is determined based on the GTV region determined by a rigid registration between the planning CT and MV-CBCT images. Each tumor template is produced for a treatment course by cropping the GTV region, which is first enhanced by a Sobel filter for edge enhancement or by a blob structure enhancement (BSE) filter for tumor enhancement. The tumor location is then estimated by the centroid of the GTV region in the MV-CBCT image using a tumor-template matching technique.

### 2.2. A Test Phantom

A lung screening phantom for CT (LSCT-001 type phantom, Kyoto Kagaku Co., Ltd., Kyoto, Japan) was employed for testing the proposed framework as validation test. Simulated tumors (CT value of lung: −900 HU, contrast of CT value between lung and simulated tumor: 270 HU, sphere shape, and diameter: 10 mm) were placed in apex pulmonis, tracheal bifurcation, and basis pulmonis of the lung screening phantom.

### 2.3. Clinical Cases

The institutional review board of our university hospital approved this retrospective study.  Table 1 shows the patient characteristics of the cases included in this study. We selected 10 patients (age: 71–89 years, median: 76 years, seven males, and three females) with non-small-cell lung cancer, who were treated with SBRT from March 2010 to August 2011, based on selection criteria on tumor, that is, solitary, type (only solid), location in lung, and size. Eight tumors were solitary and two tumors were close to or attached to the lung wall. Five tumors were located in upper lung, 2 in middle lung, and 3 in lower lung. The mean effective diameter of the 10 tumors was 10.43 ± 2.93 mm (range: 5.38–14.35 mm). Four MV-CBCT images of the patients were acquired for four fractions for patient positioning at a linear accelerator (ONCOR Impression plus, Siemens AG, Berlin and Munich, Germany) with an accelerating voltage of 4 MV. An MV-CBCT imaging was performed for initial patient positioning in each fraction, but not repositioning between beams. The total prescribed dose for each patient was 48 Gy (12 Gy/fraction) at the isocenter for four fractions.

### 2.4. Planning CT and CBCT Images

Planning CT images were acquired from a 64-slice CT scanner (SOMATOM Sensation 64, Siemens, Munich), whose CT images had an axial field of view (FOV) of 500 mm × 500 mm, a matrix size of 512 × 512, a pixel size of 0.9766 mm, a slice thickness of 1.024 mm, and a bit depth of 12. The number of planning CT images ranged from 156 to 224.

MV-CBCT images were taken by using a CBCT imaging system (MVision, Siemens, Munich) including an EPID (OPTIVUE 2.0, Siemens, Munich).

The CBCT imaging system collects projection images on the EPID and reconstructs the CBCT images (a FOV of 274 mm × 274 mm × 274 mm, a matrix size of 256 × 256, a pixel size of 1.0703 mm, a slice thickness of 1.0 mm, and a bit depth of 12).

**Figure 2:**
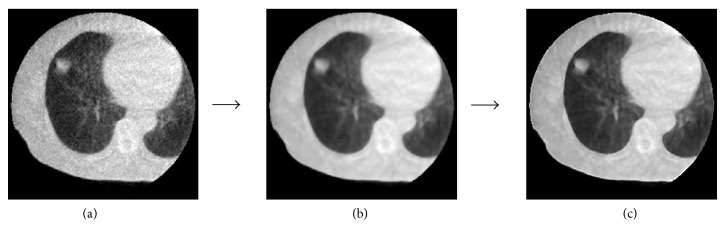
Illustrations of (a) an original MV-CBCT image, (b) a noise-reduced MV-CBCT image obtained using a median filter, and (c) an edge-enhanced MV-CBCT image obtained using a Laplacian of Gaussian (LoG) filter.

The original planning CT images and the MV-CBCT images were converted to images with a 1.0 mm isotropic voxel size using a 3D tricubic interpolation technique. The isotropic images were processed by a median filter (filter size: 3 × 3 × 3) for reducing image noise and by a Laplacian of Gaussian (LoG) filter (standard deviation: 0.7 pixels) for edge enhancement, because the MV-CBCT images had image noise and blurred target edges due to Compton scattered X-rays and high energy X-rays.  Figure 2 shows an original MV-CBCT image (a), a noise-reduced image by a median filter (b), and an edge-enhanced image by a LoG filter (c). The image noise was reduced, and the target edge was slightly enhanced.

### 2.5. Determination of a Region to Search for the Tumor Location in MV-CBCT Images

**Figure 3:**
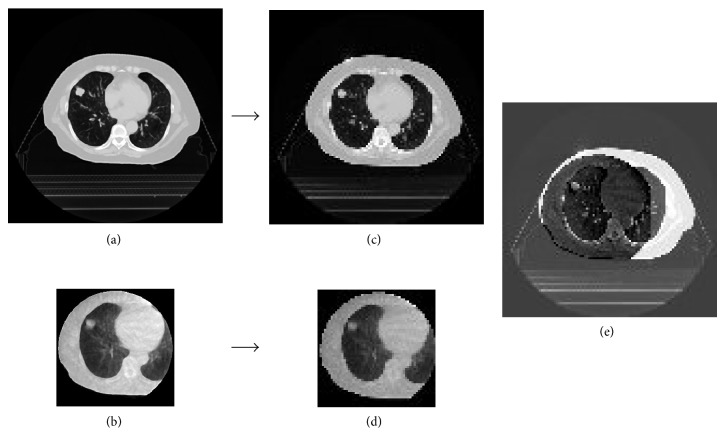
Illustrations of (a) a 1.0 mm isotropic planning CT image, (b) an MV-CBCT image, (c) a 5.0 mm isotropic planning CT image, (d) an MV-CBCT image, and (e) a subtraction image between a planning CT image and an MV-CBCT image after the rigid registration.

The region to search for the tumor location was determined based on the GTV region determined by a rigid registration between the planning CT and MV-CBCT images. First, planning CT and MV-CBCT images were transformed by using a 3D linear interpolation technique to 5.0 mm isotropic images in order to reduce the calculation time for the registration. Second, the rigid registration was performed by finding the location with the maximum cross correlation coefficient between the planning CT and MV-CBCT images according to the following equation [21]:

(1) xmax⁡,ymax⁡,zmax⁡=argmax⁡x,y,z⁡Cx,y,zCx,y,z=1LMN ×∑i=0L−1 ∑j=0M−1 ∑k=0N−1ti,j,k−t−fx+i,y+j,z+k−f−σt·σf,

where *C*(*x*, *y*, *z*) is the cross correlation coefficient at a coordinate (*x*, *y*, *z*) on a planning CT image, *L*, *M*, *N* are the numbers of pixels in the *x*, *y*, and *z* directions in an MV-CBCT image, respectively, *t*(*i*, *j*, *k*) is the pixel value at a coordinate (*i*, *j*, *k*) on an MV-CBCT image, *f*(*x*, *y*, *z*) is the pixel value at a coordinate (*x*, *y*, *z*) on a planning CT image, $\overset{-}{t}$ and *σ*_*t*_ are the mean and standard deviation of pixel values in an MV-CBCT image, and $\overset{-}{f}$ and *σ*_*f*_ are the mean and standard deviation of pixel values in a planning CT image. The ranges of *x*, *y*, and *z* coordinates, *R*_*x*_, *R*_*y*_, *R*_*z*_, are determined by the following equation:

(2) Rx=X−LRy=Y−MRz=Z−N,

where *X*, *Y*, *Z* are the numbers of pixels in the *x*, *y*, and *z* directions of a planning CT image. Please note that the cross correlation coefficient was calculated within an overlapped region between the planning CT and MV-CBCT images. The overlapped region between the planning CT and MV-CBCT images is always the same as the matrix size of the MV-CBCT image, because all MV-CBCT images are smaller than the planning CT images.  Figure 3 shows 1.0 mm isotropic planning CT (a) and MV-CBCT images (b), 5.0 mm isotropic planning CT (c) and MV-CBCT images (d), and a subtraction image (e) between the planning CT and MV-CBCT images after the rigid registration.

Third, the 5.0 mm isotropic planning CT and MV-CBCT images were converted again to 1.0 mm isotropic images using the same interpolation method. After this step, only 1.0 mm isotropic images were used for all the following processes. Fourth, the centroid of a GTV region in the MV-CBCT image was placed at the isocenter after the rigid registration. Finally, a circumscribing cuboid of the GTV region dilated by 20 pixels in each coordinate direction was derived as the region to search for the tumor location. The dilation processing was performed to 26 neighbors of a center pixel.

### 2.6. Extraction of a Tumor Template from Planning CT Images

Tumor templates were extracted by cropping a circumscribing cuboid of the GTV from the 1.0 mm original planning CT image. These templates were referred to as “planning CT tumor templates.” The coordinates of isocenters and contour data of GTVs were obtained from DICOM-RT structure sets, which were produced on a radiation treatment planning (RTP) system (XiO, Elekta, Stockholm).

### 2.7. Enhancement of the Tumor Region in Planning CT Tumor Templates and MV-CBCT Images

Planning CT tumor templates and MV-CBCT images were processed for the enhancement of tumors by using a Sobel filter or a blob structure enhancement (BSE) filter. The Sobel filter with a filter size of 3 × 3 × 3 pixels was used to enhance the tumor edges.

The BSE filter can selectively enhance spherical structures [22], such as lung tumors, based on eigenvalues of the following Hessian matrix:

(3) Hp,σ=Ixxp,σIxyp,σIxzp,σIyxp,σIyyp,σIyxp,σIzxp,σIzyp,σIzzp,σIxiyjzkp,σ=∂2∂xi∂yj∂zkGp,σ∗Ipi+j+k=2,

where *H*(**p**, *σ*) is the Hessian matrix at a position vector **p** = (*x*, *y*, *z*) and *G*(**p**, *σ*) is the Gaussian function with a standard deviation *σ*. The BSE filter was defined by

(4) Fλ1,λ2,λ3 =λ3ψλ2,λ3ψλ1,λ2if  λ3≤λ2≤λ1≤00otherwiseψλs,λt=λsλtγif  λs≤λt<00otherwise,

where *λ*_1_, *λ*_2_, *λ*_3_ were the first, second, and third eigenvalues of the Hessian matrix *H*(**p**, *σ*), respectively, *ψ* was the weight function, and *γ* was the control factor for the sharpness of selectivity for the conditions of the local structure. In this study, the standard deviation, *σ*, was set as the quarter of the effective diameter of a GTV region, and *γ* was set as 1.0. These parameters were empirically determined so that the tumors were well enhanced based on a consensus between a radiation oncologist and a medical physicist. The effective diameter, *d*, for the GTV region was calculated by the following equation:

(5) $$\begin{array}{c} d=2\sqrt[{3}]{\frac{3V_{\text{GTV}}}{4\pi }}, \end{array}$$

where *V*_GTV_ was the GTV produced on a RTP system (XiO, Elekta, Stockholm).

### 2.8. Estimation of Tumor Locations Based on a Template Matching Technique

A tumor's location was estimated based on template matching between a planning CT tumor template and an MV-CBCT image within the search region. The template matching was performed by determining the location with the maximum cross correlation coefficient between the planning CT tumor template and MV-CBCT image according to (1). After the template matching technique was applied, the GTV region was placed in the MV-CBCT image, and the centroid of the binarized GTV region was considered to be the tumor location. The binarized GTV region was obtained by using an Otsu's thresholding method [23]. The centroid (*x*_*c*_, *y*_*c*_, *z*_*c*_) of the GTV region was calculated by following equation:

(6) $$\begin{array}{c} \left(x_{c},y_{c},z_{c}\right)=\left(\frac{1}{T}\left(\overset{T}{\underset{i=0}{\sum }}x_{i}\right),\frac{1}{T}\left(\overset{T}{\underset{i=0}{\sum }}y_{i}\right),\frac{1}{T}\left(\overset{T}{\underset{i=0}{\sum }}z_{i}\right)\right), \end{array}$$

where (*x*_*i*_, *y*_*i*_, *z*_*i*_) was a coordinate of a pixel in the binarized GTV region and *T* was the number of pixels in the GTV region.

### 2.9. Evaluation of the Proposed Framework

The reference tumor region in each MV-CBCT image was determined based on a consensus between a radiation oncologist and a medical physicist. The centroid of the reference tumor region in an MV-CBCT image was considered to be the reference tumor location (grand truth). The proposed framework was evaluated by measuring location errors between the reference tumor location and the tumor location estimated by the proposed framework in three directions (left-right, anterior-posterior, and superior-interior) and its corresponding Euclidean distance. The average and the standard deviation of location errors were analyzed by using Student *t*-test and *F*-test, respectively.

## 3. Results

**Figure 4:**
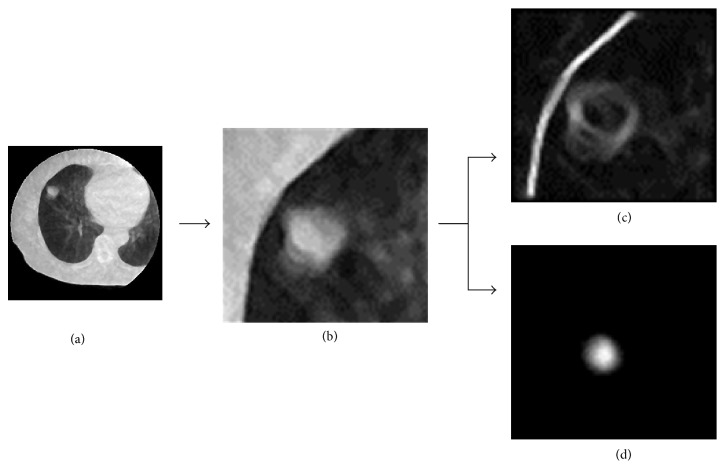
Illustrations of (a) an MV-CBCT image, (b) a search region extracted from the MV-CBCT image, (c) an edge-enhanced image obtained using the Sobel filter in the search region, and (d) a tumor-enhanced image obtained using the BSE filter in the search region.

**Figure 5:**
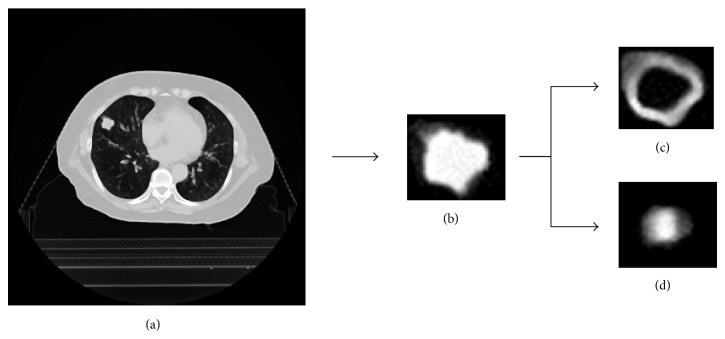
Illustrations of (a) a planning CT image, (b) a planning CT tumor template, (c) an edge-enhanced tumor template obtained using a Sobel filter, and (d) a spherical shape-enhanced tumor template obtained using a BSE filter.

Figure 4 shows an MV-CBCT image (a), a search region extracted from the MV-CBCT image (b), an edge-enhanced image with the Sobel filter in the search region (c), and a spherical shape-enhanced image obtained using the BSE filter in the search region (d).  Figure 5 shows a planning CT image (a), a planning CT tumor template (b), an edge-enhanced tumor template with a Sobel filter (c), and a spherical shape-enhanced tumor template obtained using a BSE filter (d). The tumor appearance in the MV-CBCT image was blurred due to high-energy X-rays and scattered X-rays, compared with that in the planning CT image. The degree of the tumor edge enhancement with the Sobel filter in the MV-CBCT image shown in Figure 4(c) was weaker than that in the planning CT tumor template shown in Figure 5(c). In contrast, the strength of the tumor enhancement with the BSE filter in the MV-CBCT image shown in Figure 4(d) was comparable with that in the planning CT tumor template shown in Figure 5(d).

**Figure 6:**
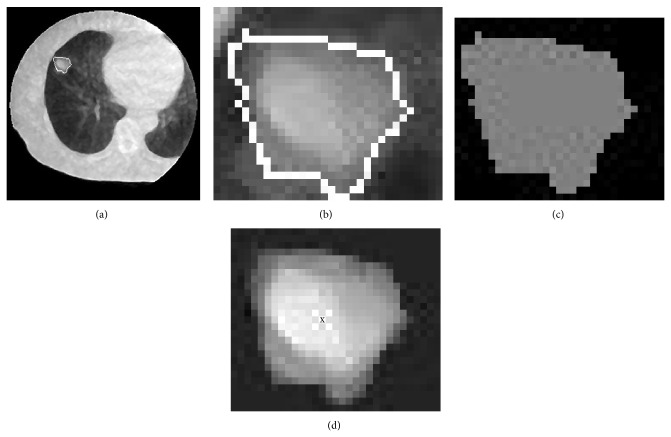
Illustrations of (a) an MV-CBCT image with a GTV region determined by the proposed framework, (b) a GTV region cropped from an MV-CBCT image, (c) a binarized GTV region indicated in gray, and (d) a tumor location indicated by x.

Figure 6 shows an MV-CBCT image with a GTV region determined by the proposed framework (a), an original GTV region and its outline (white solid line) cropped from an MV-CBCT image (b), a binarized GTV region indicated in gray (c), and the original GTV region with an estimated tumor location indicated by x (d). The tumor location indicated by x (d) was estimated as the centroid of the GTV region indicated in gray (c), as shown in Figure 6.

According to the results of the phantom study, the average Euclidean distances of the location errors in the original image, Sobel-filtered images, and BSE-filtered images were 2.0 ± 4.1 mm, 12.8 ± 9.4 mm, and 0.4 ± 0.5 mm, respectively. The proposed framework using the BSE filter can estimate the tumor locations with the errors less than 1 mm in all locations in the lung.

**Figure 7:**
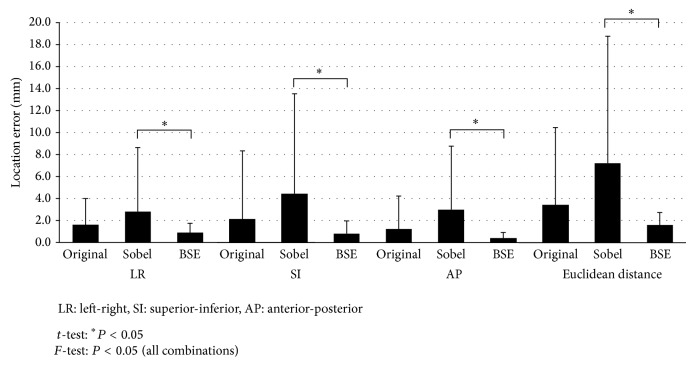
The results of a comparison of the location errors estimated by the proposed framework using three different types of planning CT tumor templates, that is, original images, Sobel-filtered images, and BSE-filtered images.

Figure 7 shows the location errors estimated by the proposed framework using three different types of planning CT tumor templates, that is, original images, Sobel-filtered images, and BSE-filtered images. The average Euclidean distances of the location errors in the original image templates, Sobel-filtered templates, and BSE-filtered templates were 3.4 ± 7.1 mm, 7.2 ± 11.6 mm, and 1.6 ± 1.2 mm, respectively. The means of the location errors with the two filtered templates had no statistically significant difference with the original image templates (*P* > 0.05). However, the estimation of the tumor locations obtained using BSE filtered templates was significantly more accurate on average compared with the Sobel-filtered templates (*P* < 0.05). In addition, the standard deviations of the location errors with the BSE-filtered templates were significantly smallest in all three directions and the Euclidean distance among the three templates (*P* < 0.05). These results on the standard deviations of the location errors mean that the precision of the proposed framework using the BSE-filtered templates was highest among the three templates.

## 4. Discussion

Guckenberger et al. reported that the intraobserver and interobserver variability of the subjective evaluation of lung tumor location errors were 0.9 ± 0.8 mm (maximum 3.5 mm) and 2.3 ± 1.1 mm (maximum 4.4 mm), respectively, in SBRT when using a CBCT-based IGRT [24]. On the other hand, the average Euclidean distance of tumor errors in the lung screening phantom was 4.84 ± 1.45 mm, which was measured by using an image guided patient positioning (IGPP) system in the radiotherapy system used for this study. The location errors (1.6 ± 1.2 mm) obtained by the proposed framework based on the BSE filter were therefore smaller than those by the IGPP system as well as the interobserver variability, but larger than the intraobserver variability.

The systematic and random errors [25, 26] of manual patient positioning measured for 36 lung cancer patients, which were chosen based on the same selection criteria as the 10 test cases used for this study, were 3.53 mm and 4.01 mm, respectively. On the other hand, the accuracy and precision of the proposed automated framework were 1.6 mm and 1.2 mm, respectively, which were smaller than those in the manual method, although the accuracy and precision of manual patient positioning depend on the institution.

In principle, the proposed framework can be applied to kV-CBCT as well as MV-CBCT. The soft tissue contrast in kV-CBCT images is higher than that in MV-CBCT images [27]. Therefore, the results obtained using kV-CBCT images may be better than the results obtained using MV-CBCT images. Furthermore, additional patient dose at the kV-CBCT imaging could be less than that at the MV-CBCT imaging, because the mean dose of the simulated tumor in the lung screening phantom was 5.8 cGy at the MV-CBCT imaging used in this study, which was larger than D50 of 0.2 to 0.42 cGy to the heart for representative thorax images at the kV-CBCT imaging reported by Ding and Munro [28].

The proposed framework has two major limitations: the calculation time and the limited types of cases. The average and maximum processing time of the proposed framework were 40 minutes and 58 seconds and 72 minutes and 12 seconds, respectively. This limitation may be overcome by implementing the proposed framework with general purpose computing on graphics processing units (GPGPU) or parallel computing techniques on computer clusters. The second limitation is that the proposed framework was applied only to solid lung cancer cases. Therefore, the proposed framework needs to be applied to large databases, including cases with various types of lung cancers, such as ground-glass opacity (GGO), and cases with diseases such as pneumonia, in order to determine whether it can also be applicable to such cases.

## 5. Conclusion

We have proposed an automated framework for estimating the lung tumor locations for tumor-based patient positioning with MV-CBCT during SBRT. The proposed framework with the BSE filter could automatically estimate the lung tumor locations with errors less than 1 mm for a lung screening phantom and 2 mm for clinical cases. If the calculation time of the proposed framework was improved, the proposed framework based on the BSE filtered image might be one of the tools for tumor-based patient positioning in SBRT.

## Figures and Tables

**Table 1:** Patient characteristics for 10 cases used in this study.

Case number	Age	Gender^*^	Tumor location	Number of fractions	Effective diameter (mm)
1	89	M	Lt. upper	4	9.35
2	75	M	Lt. middle	4	12.30
3	72	F	Rt. middle	4	11.94
4	83	M	Rt. upper	4	10.07
5	80	M	Rt. upper	4	12.91
6	66	M	Rt. lower	4	14.35
7	71	M	Rt. lower	4	5.90
8	75	F	Lt. upper	4	5.38
9	83	F	Rt. upper	4	9.00
10	77	M	Rt. lower	4	13.12

^*^F: female and M: male.
